# Supplementary material for: Navigating public, semi-public, and private drug use environments: A qualitative examination of unsheltered homelessness and drug use in Baltimore City
Source: PLoS One. 2025 Dec 3;20(12):e0338107. doi: 10.1371/journal.pone.0338107 (PMC12674580; doi:10.1371/journal.pone.0338107)
Supplement: Attachment — Submitted filename: ReponseToReviewers_28Aug2025.docx [file pone.0338107.s003.docx]

**Response to Reviewers for “Navigating public, semi-public, and private drug use environments: A qualitative examination of unsheltered homelessness and drug use in Baltimore City” [PONE-D-25-23824]**

Dear Dr. Mitra,

Thank you for the opportunity to revise this manuscript. We thank the reviewers for their thoughtful comments and have responded to each comment, as outlined below.

**COMMENTS FOR THE AUTHOR:**

**From the Editor –**

1. Please ensure that your manuscript meets PLOS ONE's style requirements, including those for file naming. The PLOS ONE style templates can be found at <https://journals.plos.org/plosone/s/file?id=wjVg/PLOSOne_formatting_sample_main_body.pdf> and <https://journals.plos.org/plosone/s/file?id=ba62/PLOSOne_formatting_sample_title_authors_affiliations.pdf>

RESPONSE: The manuscript has been revised to meet PLOS ONE’s style requirements. Appropriate level headings have been applied to the manuscript sections. The naming convention for Figure 1 in line 170 on page 9 of the unmarked revised manuscript has been revised to “Fig 1”. The table 1 formatting has been updated to meet the style requirements. In-text citations have been updated to cite references in brackets. Funding information has been removed from the acknowledgements, and the following statement has been added in the Acknowledgements section in lines 671-672 on page 30 of the unmarked revised manuscript: “The authors would like to acknowledge ALIVE study staff who assisted with recruitment and thank the research participants who shared their experiences and trusted us with their stories.”

2. Thank you for stating the following financial disclosure:

[This study was funded by the National Institute on Drug Abuse [U01DA036297, PIs: Mehta/Kirk; R01DA053136, PI: Genberg], the Johns Hopkins Bloomberg School of Public Health Center for Qualitative Studies in Health and Medicine’s Dissertation Enhancement Award, and The Paul V. Lemkau Scholarship Fund. AQT was supported by the National Institute on Drug Abuse [T32DA007292-31A1; PIs: Johnson/Maher].].

Please state what role the funders took in the study. If the funders had no role, please state: "The funders had no role in study design, data collection and analysis, decision to publish, or preparation of the manuscript."

If this statement is not correct you must amend it as needed.

Please include this amended Role of Funder statement in your cover letter; we will change the online submission form on your behalf.

RESPONSE: The funders had no role in the study design, data collection and analysis, decision to publish, or preparation of the manuscript. This statement has been added to the final paragraph of our cover letter.

3. We note that you have indicated that there are restrictions to data sharing for this study. For studies involving human research participant data or other sensitive data, we encourage authors to share de-identified or anonymized data. However, when data cannot be publicly shared for ethical reasons, we allow authors to make their data sets available upon request. For information on unacceptable data access restrictions, please see http://journals.plos.org/plosone/s/data-availability#loc-unacceptable-data-access-restrictions.

Before we proceed with your manuscript, please address the following prompts:

a) If there are ethical or legal restrictions on sharing a de-identified data set, please explain them in detail (e.g., data contain potentially identifying or sensitive patient information, data are owned by a third-party organization, etc.) and who has imposed them (e.g., a Research Ethics Committee or Institutional Review Board, etc.). Please also provide contact information for a data access committee, ethics committee, or other institutional body to which data requests may be sent.

b) If there are no restrictions, please upload the minimal anonymized data set necessary to replicate your study findings to a stable, public repository and provide us with the relevant URLs, DOIs, or accession numbers. Please see http://www.bmj.com/content/340/bmj.c181.long for guidelines on how to de-identify and prepare clinical data for publication. For a list of recommended repositories, please see https://journals.plos.org/plosone/s/recommended-repositories. You also have the option of uploading the data as Supporting Information files, but we would recommend depositing data directly to a data repository if possible.

Please update your Data Availability statement in the submission form accordingly.

RESPONSE: There are ethical restrictions on sharing de-identified data from this study because the data contains potentially identifying and sensitive patient information. These restrictions are imposed by the Johns Hopkins Institutional Review Board. De-identified data requests may be sent to the ALIVE study PIs, Shruti Mehta ([smehta@jhu.edu](mailto:smehta@jhu.edu)) and Gregory Kirk ([gdk@jhu.edu](mailto:gdk@jhu.edu)), or the Johns Hopkins Bloomberg School of Public Health IRB office (BSPH.irboffice@jhu.edu). The Data Availability statement has been updated accordingly.

4. Please include a separate caption for each figure in your manuscript.

RESPONSE: A separate caption has been added for Figure 1 in line 177 on page 9 of the unmarked revised manuscript.

5. Please review your reference list to ensure that it is complete and correct. If you have cited papers that have been retracted, please include the rationale for doing so in the manuscript text, or remove these references and replace them with relevant current references. Any changes to the reference list should be mentioned in the rebuttal letter that accompanies your revised manuscript. If you need to cite a retracted article, indicate the article’s retracted status in the References list and also include a citation and full reference for the retraction notice.

RESPONSE: We have reviewed the reference list and confirm it is complete and correct.

**From the Reviewers –**

**Reviewer 1:**

1. Well written article- I have attached the PDF of suggested edits.

I have also included other limitations that need to be added –

1) Small sample size

2) Participants were drawn from the AIDS Linked to Intravenous Experience (ALIVE) Study, an ongoing cohort study. This could result in a selection bias. as being involved in long-term research may differ from those who are not engaged with such services or institutions.

3) The study relies on self report. Probable biases include recall bias, and underreporting of risky behaviors due to stigma or legal fears.

4) The study relies only on qualitative interview data, without integrating observational data or input from service providers, which could have strengthened the findings.

RESPONSE: Thank you for including suggested edits in the PDF. I have transferred the suggested edits and line numbers in this document, as well as included our response to the suggested edits.

2. Line 33: Sample size seems to be too small at 9.

RESPONSE: Thank you for your comment. This study was conceptualized and carried out using interpretative phenomenological analysis (IPA). Lines 131-134 on page 7 of the unmarked revised manuscript have been edited to convey the justification for small sample sizes in IPA studies: “According to Smith et al., sample sizes in studies using IPA are necessarily small due to the approach’s focus on understanding the particularities of participants’ daily lived experiences, and to enable deep analysis of each interview [52]. As such, larger sample sizes are typically discouraged in IPA studies, and recommended sample sizes range from 4-10 [53, 54].”

Due to this study’s use of IPA methodology, we believe that the sample size of 9 is appropriate for the qualitative method used in this study and sufficient to capture the phenomenon under study while also allowing for a detailed analysis of the particularities in each participant’s lived experiences of homelessness and drug use.

3. Lines 120-124: The small sample size significantly reduces the power of the study.  This makes sense for an IPA study, however would have loved to see a higher sample size of around 30-35 which could have definitely have been done given the large size of the population being studies here.

RESPONSE: Given the parent study we sampled participants from, it was likely possible to sample a larger number of individuals who were experiencing unsheltered homelessness and using drugs. However, we employed sampling procedures that were in line with IPA because our study was conceptualized and carried out using this method [1, 2]. As specified in response to your second comment, small sample sizes are necessary in IPA studies to enable a detailed analysis of the nuances in participants’ daily lived experiences around the phenomenon under study. Larger sample sizes are typically discouraged in IPA studies, as they do not facilitate detailed analyses of participants’ experiences.

4. Line 136: An objective drug testing would have been a more reliable and objective method.

RESPONSE: Thank you for your comment. While we recognize that drug testing enables the detection of drugs in an individual’s system, we chose to rely on self-reported drug use for a number of reasons. First, as noted in line 156 on page 8 of the unmarked revised manuscript, we asked about their drug use in the past 30 days during the eligibility screening. During the interview, participants spoke extensively about their drug use experiences and were probed to speak in further detail about those experiences. Thus, our concern about the participant misremembering or forgetting their drug use was minimized. Second, we recruited participants from the AIDS Linked to IntraVenous Experience Study, which has been ongoing for over 30 years. ALIVE staff have established strong rapport and mutual trust with participants over this period. Recruiting participants through the ALIVE study and with support from ALIVE staff created a trusting environment for participants to more openly and comfortably discuss sensitive topics, such as their drug use. Third, constructivism is the epistemology that underlies qualitative research. From the constructivist lens, knowledge is co-created by the researcher and the participant, and what a participant expresses to the researcher is perceived as the participant’s reality or “truth.” In contrast, objectivism is the epistemology that underlies quantitative research. Objectivism prioritizes being “objective” and reducing bias. Therefore, due to this study’s use of qualitative methods, we believed asking participants to self-report their drug use was appropriate and sufficient to determine their eligibility in the study. Fifth, the use of self-reported drug use is in line with what previous qualitative studies with people who use drugs have used to determine eligibility, including qualitative studies that have recruited from the ALIVE study [3-6].

5. Line 615: Limitations to be included-

1) Small sample size

2) Participants were drawn from the AIDS Linked to Intravenous Experience (ALIVE) Study, an ongoing cohort study. This could result in a selection bias. as being involved in long-term research may differ from those who are not engaged with such services or institutions.

3) The study relies on self report. Probable biases include recall bias, and underreporting of risky behaviors due to stigma or legal fears.

4) The study relies only on qualitative interview data, without integrating observational data or input from service providers, which could have strengthened the findings.

RESPONSE: Thank you for your suggestions on additional limitations to be included. Below are our responses to each of the limitations you identified.

Limitation 1

As indicated in our responses to your second and third comments related to the study’s sample size, we have addressed your concerns by including additional rationale for the sample size used and why the sample size is appropriate for an IPA study in lines 131-134 of the unmarked revised manuscript. IPA studies recommend sample sizes of 4-10 [1, 2].

Limitations 2 and 3

We have incorporated your second and third suggested limitations in lines 637-660 on pages 28-29 of the unmarked revised manuscript. In the manuscript, we discussed your proposed second and third limitations using the trustworthiness criteria for qualitative studies, which is often used to measure rigor in qualitative studies [7, 8]. Traditional scientific criteria applied in quantitative studies (i.e., internal validity, external validity or generalizability, reliability, objectivity) is not well-suited for qualitative studies due to differences in philosophical underpinnings of what knowledge is and how it is obtained in qualitative and quantitative research [8]. In other words, there are differences in the epistemology of qualitative research (i.e., constructivism) and epistemology of quantitative research (i.e., objectivism) that make trustworthiness criteria a more suitable indication of rigor in qualitative research. According to the trustworthiness criteria, credibility is analogous to internal validity, transferability is analogous to external validity, dependability is analogous to reliability, and confirmability is analogous to objectivity [7, 8]. We address your second concern about selection bias as a limitation of the transferability of our findings in lines 637-641 of the unmarked revised manuscript: “Participants were also recruited from a prospective cohort study of people with a history of injection drug use, whose engagement with the study may be a marker of increased engagement in, or referral to, health services and resources. Therefore, the transferability of findings may be limited to individuals who use drugs and live in similar urban contexts in the US, and those who have greater engagement with health and social services.” We address your third concern about recall bias and underreporting as a limitation of the credibility of our findings in lines 644-660 of the unmarked revised manuscript: “Drug use and homelessness are highly sensitive topics; some participants may have been hesitant to speak openly about their experiences due to stigma and fear of legal repercussions. Our study also did not formally conduct member checking but completed respondent validation during the data collection process. Therefore, some dimensions of drug use and unsheltered homelessness may have been missed during data collection and data analysis, affecting the credibility of our findings. However, the ALIVE study has been ongoing for over 30 years, and ALIVE staff have established rapport and mutual trust with participants—thereby fostering a destigmatizing and comfortable environment for individuals to participate in research studies. During interviews, participants demonstrated comfort in speaking at length and sharing extensive detail about their experiences with drug use and homelessness. While [authors blinded for review] do not have personal lived experience with homelessness and drug use, they are informed “outsiders” with backgrounds in substance use and public health and experience working with socially marginalized populations, including unsheltered populations. Being an informed “outsider” may have also encouraged participants to describe their experiences in detail during their interviews, with the assumption that [author blinded for review] is not knowledgeable on the lived realities of unsheltered PEH who use drugs.”

Limitation 4

Although this qualitative study filled a gap in research on the drug use experiences of people who are unsheltered by gathering perspectives of people with lived experience, it was beyond the scope of this study to understand perspectives of providers from behavioral health and housing sectors. Insight from these groups could have identified potential structural and programmatic aspects of drug use and overdose experiences that may not have otherwise been understood by exploring the perspectives of people with lived experience. Likewise, while this study did not have a multi-method design, future quantitative analysis can generate empirical evidence on relationships of risk environment features (e.g., policing) and engagement in harm reduction practices with overdose.

**Reviewer 2:**

1. Line 26: Due to their economic and social marginalization. Does social margination includes economic?

RESPONSE: Thank you for this question. In the abstract, we name economic and social marginalization separately because we conceptualize social marginalization in this context to encompass the exclusion or restriction of people experiencing homelessness from relationships and interactions with others. In contrast, we conceptualize economic marginalization in this context to be the exclusion or restriction of people experiencing homelessness from access to income or income-generating activities and resources.

2. Line 31: Please define public, semi-public, and private places

RESPONSE: We have added the requested definitions in lines 40-42 of the unmarked revised manuscript: “Public settings (e.g., street) are accessible to the general public, while semi-public settings (e.g., library restrooms) provide public access with some restrictions. In contrast, private settings (e.g., private residence) are under private ownership with no public access.”

3. Line 32: AIDS Linked to Intravenous Experience Study “(ALIVE)”

RESPONSE: The sentence has been updated to include the study’s acronym on line 43 of the unmarked revised manuscript.

4. Line 81: people who inject drugs (PWID)

RESPONSE: We have changed line 93 in the unmarked revised manuscript from “PWUD” to “people who inject drugs (PWID).”

5. Line 72: and temporarily/transitionally housed. Refer to line 114/5, which divides into unsheltered, sheltered, and transitional housed.

RESPONSE: Lines 114-115 of the unrevised manuscript describe the percentage of people experiencing homelessness in Baltimore City who reside in emergency shelters, transitional housing, and unsheltered contexts. Based on the US Department of Housing and Urban Development’s definition of literal homelessness, which this study uses, those who reside in emergency shelters and in transitional housing are defined as experiencing sheltered homelessness. We have revised lines 124-127 on page 7 of the unmarked revised manuscript to align with the definitions of sheltered and unsheltered homelessness: “According to the 2023 Baltimore City Point-in-Time (PIT) Count, approximately 92% of the 1,550 PEH reported experiencing sheltered homelessness (including residing in emergency shelters and transitional housing), and less than 7% were unsheltered (51).”

6. Line 123: 3 people were excluded due to mental health- tell us more because substance abuse is also a mental health disorder

RESPONSE: Thank you for asking us to clarify this. While re-reviewing the sentence, we identified a typo. The sentence is missing the word “or” and has been revised in lines 135-137 on page 7 of the unmarked revised manuscript to be: “Although we conducted interviews with 12 participants, three were excluded from the analysis because they were ended early or due to concerns about the participants’ mental health during the interview.” We have also updated the manuscript to include an explanation of why each interview was excluded in lines 137-141 on page 7 of the unmarked revised manuscript: “Specifically, two interviews were ended early because one participant was too lethargic to continue and one participant was losing her voice and what she said could not be clearly heard. A third interview was excluded because the participant expressed paranoia and what he discussed was consequently difficult to understand.”

7. Line 127: speedball (cocaine + opioid?)

RESPONSE: We have clarified lines 144-145 on page 8 of the unmarked revised manuscript to specify that speedball refers to the use of cocaine and heroin together: “…(e.g., cocaine alone, heroin alone, “speedball” which refers to use of cocaine and heroin together…”

8. Line 130: Why were sheltered people excluded, because this study focused on unsheltered with PEH?

RESPONSE: People experiencing sheltered homelessness were not included because this study sought to fill a gap in the literature on the drug use environment and experiences of people experiencing unsheltered homelessness. As noted in lines 105-109 on page 6 of the unmarked revised manuscript: “…despite research documenting that risk factors (e.g., police interactions) in micro-level drug use environments may be exacerbated for unsheltered PEH who only have access to visible and public and semi-public settings compared to sheltered PEH and the general population, there is a dearth of studies qualitatively examining the intersecting phenomena of unsheltered homelessness and drug use [17, 44].” We also refer to people experiencing unsheltered homelessness as unsheltered people experiencing homelessness (PEH) or unsheltered PEH in the manuscript. Additionally, in qualitative studies, it is recommended to sample individuals who are very familiar with and can speak in detail about the phenomenon being examined. Therefore, we believe it was most appropriate to interview people experiencing unsheltered homelessness due to the study’s aim. In other words, people experiencing sheltered homelessness have different sleeping environments and drug use space. As a result, they would not be able to speak to the unique experiences of being unsheltered.

9. Line 423: Why is part of the conversation in quotes?

RESPONSE: There are quotation marks around part of the participant’s response because he is quoting what another person may say to him.

**References**

1. Noon EJ. Interpretive phenomenological analysis: An appropriate methodology for educational research? Journal of perspectives in applied academic practice. 2018;6(1):75-83.

2. Smith J, Flower P, Larkin M. Interpretative phenomenological analysis: Theory, method and research: SAGE Publications Inc.; 2022.

3. Patel EU, Grieb SM, Winiker AK, Ching J, Schluth CG, Mehta SH, et al. Structural and social changes due to the COVID-19 pandemic and their impact on engagement in substance use disorder treatment services: a qualitative study among people with a recent history of injection drug use in Baltimore, Maryland. Harm reduction journal. 2024;21(1):91.

4. Winiker AK, Patel EU, Genberg BL, Ching J, Schluth C, Mehta SH, et al. “Especially Being Homeless, They Just Think You’re Infected with COVID or Something”: A Qualitative Exploration of the COVID-19 Pandemic’s Impact on People Experiencing Homelessness With a History of Injection Drug Use in Baltimore, Maryland. Housing Policy Debate. 2024:1-21.

5. Winiker AK, Tobin KE, Gicquelais RE, Owczarzak J, Latkin C. “When you're getting high… you just don't want to be around anybody.” A qualitative exploration of reasons for injecting alone: perspectives from young people who inject drugs. Substance use & misuse. 2020;55(13):2079-86.

6. Sisson LN, Winiker AK, Triece T, Rousch RS, Rouhani S, Owczarzak J, et al. “All You Can Do is What You Know to Do”: Naloxone Knowledge and Uncertainty Among People Who Use Drugs in Maryland amid a Volatile Drug Market. Substance Use & Misuse. 2025;60(8):1164-72.

7. Nowell LS, Norris JM, White DE, Moules NJ. Thematic analysis: Striving to meet the trustworthiness criteria. International journal of qualitative methods. 2017;16(1):1609406917733847.

8. Lincoln Y, Guba E. Naturalistic inquiry: Sage; 1985.
